# Supplementary material for: Efficient lateral-structure perovskite single crystal solar cells with high operational stability
Source: Nat Commun. 2020 Jan 14;11:274. doi: 10.1038/s41467-019-13998-2 (PMC6959261; doi:10.1038/s41467-019-13998-2)
Supplement: Supplementary file 1 — Supplementary Information [file 41467_2019_13998_MOESM1_ESM.pdf]

Supplementary Information for

Efficient Lateral-structure Perovskite Single Crystal Solar Cells with High Operational Stability

Yilong Song[1], *Weihui Bi*[1], *Anran Wang*[1], *Xiaoting Liu*[1], *Yifei Kang*[1], *Qingfeng Dong*[1]*

[1] State Key Laboratory of Supramolecular Structure and Materials, College of Chemistry, Jilin University, Changchun, China

* Correspondence to QD, Email: qfdong@jlu.edu.cn

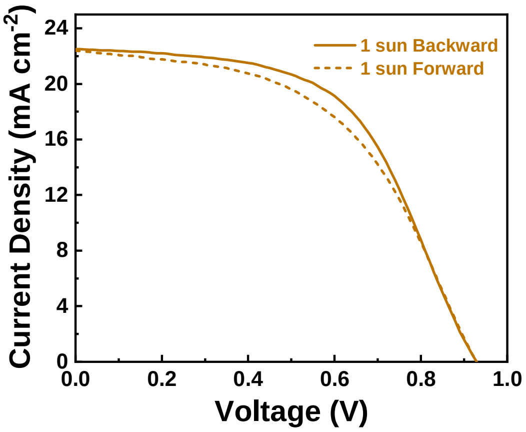

Supplementary Figure 1. The *J-V* plots of lateral structured device in both forward and backward direction under 1 Sun.

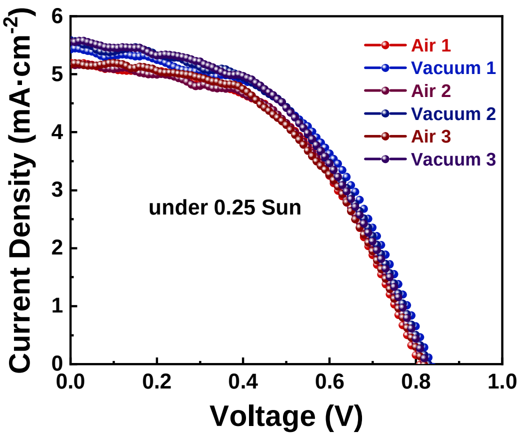

Supplementary Figure 2. The *J*-*V* curves measured in vacuum and air condition.

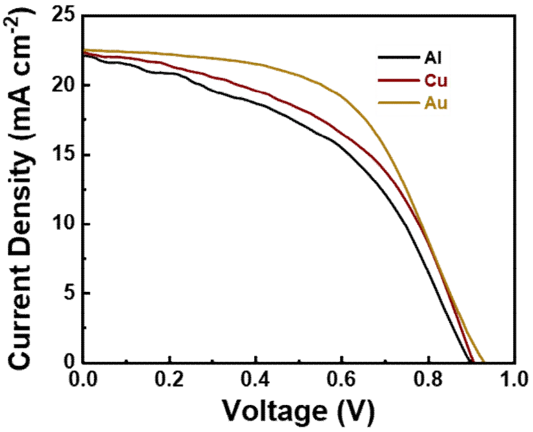

Supplementary Figure 3. The *J-V* of lateral structured device with Al, Cu or Au as cathode.

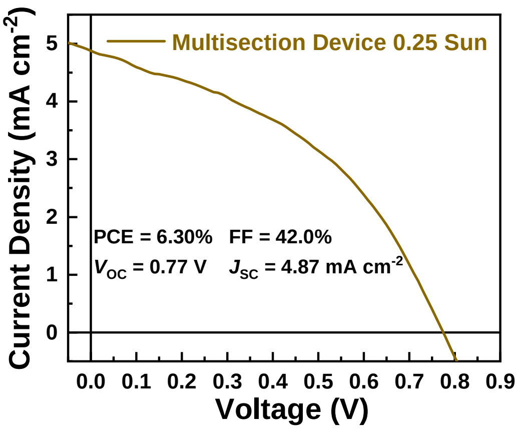

Supplementary Figure 4. The *J*-*V* curve of the interdigital lateral-structure $MAPbI_3$ single-crystalline device.
