# Supplementary material for: Efficient lateral-structure perovskite single crystal solar cells with high operational stability
Source: Nat Commun. 2020 Jan 14;11:274. doi: 10.1038/s41467-019-13998-2 (PMC6959261; doi:10.1038/s41467-019-13998-2)
Supplement: Supplementary file 3 — Solar Cells Reporting Summary [file 41467_2019_13998_MOESM3_ESM.pdf]

nature research

Corresponding author(s): Qingfeng Dong

Solar Cells Reporting Summary

Nature Research wishes to improve the reproducibility of the work that we publish. This form is intended for publication with all accepted papers reporting the characterization of photovoltaic devices and provides structure for consistency and transparency in reporting. Some list items might not apply to an individual manuscript, but all fields must be completed for clarity.

For further information on Nature Research policies, including our data availability policy, see Authors & Referees.

ü Experimental design

Please check: are the following details reported in the manuscript?

1. Dimensions

Item	Yes/No	Details
Area of the tested solar cells	[x] Yes [ ] No	For the single device area was 0.05 mm2, and for the multisection device area was 1.85 mm2. The information can be found in the Material and Device Characterization.
Method used to determine the device area	[x] Yes [ ] No	Defined by the area between anode and cathode.

2. Current-voltage characterization

Item	Yes/No	Details
Current density-voltage (J-V) plots in both forward and backward direction	[x] Yes [ ] No	Supplementary Figure 1
Voltage scan conditions<br>*For instance: scan direction, speed, dwell times*	[x] Yes [ ] No	Reverse scan, 0.03 V/s, Dwell time: 0.333 s
Test environment<br>*For instance: characterization temperature, in air or in glove box*	[x] Yes [ ] No	Devices are measured in vacuum, air and glove box at room temperature.
Protocol for preconditioning of the device before its characterization	[ ] Yes [x] No	No preconditioning is required before characterization.
Stability of the J-V characteristic<br>*Verified with time evolution of the maximum power point or with the photocurrent at maximum power point; see ref. 7 for details.*	[x] Yes [ ] No	Photocurrent output are measured by tracking the maximum power point (Figure 6g).

3. Hysteresis or any other unusual behaviour

Item	Yes/No	Details
Description of the unusual behaviour observed during the characterization	[x] Yes [ ] No	There was weak hysterisis between backward scan and forward scan.
Related experimental data	[x] Yes [ ] No	Supplementary Figure 1

4. Efficiency

Item	Yes/No	Details
External quantum efficiency (EQE) or incident photons to current efficiency (IPCE)	[ ] Yes [x] No	The light intensity in EQE measurement is too low to generate stable photo current in microscale lateral structured device.
A comparison between the integrated response under the standard reference spectrum and the response measure under the simulator	[ ] Yes [x] No	None liner photoresponse.
For tandem solar cells, the bias illumination and bias voltage used for each subcell	[ ] Yes [x] No	Single crystal perovskite solar cell is investigated in this work are not tandem solar cells.

5. Calibration

Item	Yes/No	Details
Light source and reference cell or sensor used for the characterization	[x] Yes [ ] No	Simulated AM 1.5 G irradiation was produced by a Xenon lamp solar simulator (SOLARBEAM-02-3A, CROWNTECH.INC.). The light intensity was calibrated by a reference solar cell (SRC-1000-TC-K-QZ-C, VLSI Standards).

Confirmation that the reference cell was calibrated and certified

☒ Yes ☐ No

The reference silicon detector (SRC-1000-TC-K-QZ-C, VLSI Standards) was calibrated and NREL traceable.

Calculation of spectral mismatch between the reference cell and the devices under test

☐ Yes ☒ No

No calculation was used.

6. Mask/aperture

Size of the mask/aperture used during testing

☒ Yes ☐ No

The information can be found in the Material and Device Characterization.

Variation of the measured short-circuit current density with the mask/aperture area

☐ Yes ☒ No

The efficiency distribution is provided in Figure 6c, d, e, f.

7. Performance certification

Identity of the independent certification laboratory that confirmed the photovoltaic performance

☐ Yes ☒ No

No certification

A copy of any certificate(s)
Provide in Supplementary Information

☐ Yes ☒ No

No certification

8. Statistics

Number of solar cells tested

☒ Yes ☐ No

A total of 20 single crystal perovskite solar cells were tested.

Statistical analysis of the device performance

☒ Yes ☐ No

Figure 6e and 6f.

9. Long-term stability analysis

Type of analysis, bias conditions and environmental conditions
For instance: illumination type, temperature, atmosphere humidity, encapsulation method, preconditioning temperature

☒ Yes ☐ No

in Figure 6g, the single crystal perovskite solar cell device was measured by tracking the photocurrent at its MPP condition under 1 Sun illumination
